# Coastal complexity: Ancient human diets inferred from Bayesian stable isotope mixing models and a primate analogue

**DOI:** 10.1371/journal.pone.0209411

**Published:** 2018-12-20

**Authors:** Matthew C. Lewis, Judith C. Sealy

**Affiliations:** 1 Department of Archaeology, University of Cape Town, Cape Town, South Africa; 2 Institute for Communities and Wildlife in Africa, Department of Biological Sciences, University of Cape Town, Cape Town, South Africa; 3 Department of Biological Sciences, University of Cape Town, Cape Town, South Africa; University of Hyogo, JAPAN

## Abstract

An extensive ecological literature applies stable isotope mixing models to derive quantitative dietary reconstructions from isotope ratios of consumer tissues. While this approach works well for some organisms, it is challenging for consumers with complex, varied diets, including humans; indeed, many archaeologists have avoided the use of mixing models because uncertainties in model outputs are sufficiently large that the findings are not helpful in understanding ancient lifeways. Here, we exploit an unparalleled opportunity to evaluate the feasibility of dietary quantification in a nutritionally and isotopically complex context on the Cape Peninsula, South Africa. Delta values (δ^13^C and δ^15^N) of 213 indigenous food samples enable us to characterise four food groups: terrestrial plants, terrestrial vertebrates, marine invertebrates and marine vertebrates. A recent study of baboons that consumed marine and terrestrial foods provides insight into the relationship between such foods and consumer tissue isotopes. We use this information to refine our interpretation of δ^15^N and especially δ^13^C in bone collagen from 35 archaeological hunter-gatherers, achieving better estimates of the relative importance of marine and terrestrial foods in the diet than has hitherto been possible. Based on Bayesian stable isotope mixing model (SIMM) outputs, we infer that the trophic enrichment factor (TEF) for δ^13^C_bone collagen_ in these coastal humans is closer to +3 than +5‰. In the most ^13^C- and ^15^N-rich individuals, 65–98% of bone collagen (95% credible intervals) derived from marine foods. Conversely, in ^13^C and ^15^N-poor individuals, 7–44% of bone collagen derived from marine foods. The uncertainties discussed here highlight the need for caution when implementing SIMMs in studies of consumers with complex diets. To our knowledge, this work constitutes the most detailed and most tightly constrained study of this problem to date.

## Introduction

Since stable isotope measurements of consumer tissues were first used as a tool for dietary reconstruction in the late 1970s [1–3], researchers have sought to use this approach to quantify the contributions of different (isotopically distinct) foods to diets. This has been achieved to varying degrees, with greater success in animal- than human studies (e.g. [4–8]). Humans and other omnivores are especially challenging in this regard because they consume very varied diets, in which foods may have different nutrient compositions as well as different isotope ratios. Here, we explore the value of a Bayesian stable isotope mixing model for reconstructing the contributions of marine and C_3_-based terrestrial foods to the diets of Late Holocene coastal hunter-gatherers at the south-western tip of Africa. This effort is informed by a detailed observational and isotopic (δ^13^C and δ^15^N) study of contemporary wild-foraging chacma baboons [9,10] which live in the same area and consume many of the same resources, including both terrestrial and marine foods [9–13].

The implications extend far beyond this case study. Similar (marine/terrestrial C_3_) isotopic patterning occurs in many parts of the world, including much of Europe and the Pacific, leading to debates about the relative importance of marine and terrestrial foods in a range of ancient societies [14–22]. Furthermore, calibration of radiocarbon dates measured on partly marine-derived sample materials requires estimation of the proportions of marine- and terrestrially-derived carbon [23–25].

Isotope-based studies of coastal hunter-gatherer diets in South Africa began several decades ago [26–30]. Evidence from food waste recovered through archaeological excavations provides a guide to the types of foods consumed, but differential preservation of hard and soft food remains makes it very difficult to quantify diets on this basis. Nonetheless, we know that shellfish, stranded marine mammals, fish, terrestrial vertebrates and edible plants were all important [31–35]. In the south-western, winter rainfall region, there are clear distinctions between the δ^13^C values of marine and terrestrial foods, with the former significantly enriched in ^13^C. There are also broad differences in the macronutrient composition of the foods, with marine foods generally higher in protein, whereas many terrestrial [plant] foods are richer in carbohydrates. As pointed out by Krueger and Sullivan [36] and Parkington [37], δ^13^C values of bone collagen are likely to reflect protein-rich foods more strongly than protein-poor foods. We do not, however, know the extent of this bias, which means that we can confidently interpret the isotope ratios of consumer tissues in only a conservative manner, inferring “strongly marine” or “strongly terrestrial” diets.

A number of studies have attempted to investigate this issue of “metabolic routing” by means of controlled feeding experiments and/or compound-specific analyses. Early controlled feeding studies suggested that bone collagen derives entirely from dietary protein [38,39], but later work showed that, while essential amino acids must come from the diet, non-essential amino acids synthesized in the consumer’s body may include a significant carbon contribution from dietary carbohydrates and lipids. It has been shown that the extent depends on the composition of the diet and the metabolic status of the consumer in question. However, the factors at work here are many and complex, and our understanding of them remains poor [40–43].

These uncertainties apply equally to other omnivorous consumers such as baboons. The baboons of the Cape Peninsula represent an appropriate [44], and potentially very useful, analogue for isotope-based studies of marine food use in archaeological humans. Non-human primates have been used as such in isotopic studies of hominin diets in other contexts [45,46], but not yet in coastal regions. Importantly, there is substantial overlap in foods eaten by local baboons and Holocene coastal hunter-gatherers in this region–diets of both include terrestrial plants and marine intertidal invertebrates [10,11,13,31,34,35]. The diet of an exclusively wild-foraging Peninsula troop that feeds on both marine and terrestrial foods (the Kanonkop troop) has recently been investigated, using behavioural observations and isotopic assessment of food sources and baboon tissue [9,10]. The respective mean (± SD) hair δ^13^C and δ^15^N values from adult male baboons were −21.6 ‰ (± 0.1) and 5.0 (± 0.3), and corresponding values from adult females were −21.8 ‰ (± 0.3) and 3.9 ‰ (± 0.2) [10]. Models incorporating these values indicated that marine foods contributed ≤ 17% of dietary protein for adult males, and ≤ 16% for adult females. Estimates of marine contributions to these baboons’ diets based on behavioural observations were 4.8% (± 8.7%) of feeding time for males and 3.3% (± 6.8%) of feeding time for females, and therefore corroborated findings based on stable isotope ratios. That study provides a unique opportunity to establish how a modest contribution of marine foods to the diet manifests in consumer tissue; an issue that has long been unresolved. We therefore use the baboon study as a secure starting point for a quantitative assessment of the isotope values of archaeological humans, for whom we have delta values but no observational information.

Isotope-based quantitative reconstructions of consumer diet involve the use of stable isotope mixing models (SIMMs)–mathematical models that provide estimates of proportional contributions of sources to a mixture, based on stable isotope ratios [47,48]. Over the last two decades, there has been an exponential increase in the use of SIMMs in published research [49], with the most common application being determining diet composition (e.g. [7,8,50–56]). The first linear mixing model used to investigate diet generated accurate estimates of dietary contributions (but see [57]), but was of use only in highly constrained systems [58], and therefore inappropriate for human diets. Frequentist SIMMs that allowed for greater flexibility in model specification were subsequently developed [59–61], but each permitted the user to address only one of the major assumptions implicit in modelling consumer diets [48]. More recently, SIMMs that allow for specification of much more complex models have been developed within the Bayesian framework [49,62,63]. Users are now able to incorporate prior knowledge of diet (derived from other data sources), different concentrations of C and N and different assimilation efficiencies for various foods, variation in discrimination factors, measurement-, source process- and mixture process error, and a correction for metabolic routing, often in conjunction, into the models [62–66]. These Bayesian SIMMs provide great explanatory power [64,65,67], but accuracy of model output is entirely dependent on that of model input [49,62,68,69]. As explained below, we opt to use the MixSIAR model [66], which allows for incorporation of all of the above-mentioned variables except a term explicitly describing metabolic routing.

The outputs of SIMMs are expressed as percentage contributions of the defined food groups. However, these values are not necessarily percentages of the food volume ingested, but may be closer to percentages of the relevant elements (C and N) contributed by the defined food groups. Dietary N comes almost entirely from protein, so in the case of N the model provides an estimate of % protein from the various defined food groups. Dietary C may come from protein, carbohydrates or lipids, each with very different metabolic pathways in the consumer, making different contributions to the chosen tissue. For C, therefore, the situation is much more complicated and the MixSIAR model does not engage with this complexity. Other models (e.g. FRUITS) attempt to deal with routing-related issues, allowing the user to incorporate values that quantify these processes [65]. Rigorous use thereof is however contingent on excellent understanding of the nature of the routing and the extent to which it takes place in the consumer in question. Little empirical data is available to inform such choices.

The overarching aim of this study is to use current isotope-based diet modelling techniques and isotopic and behavioural data regarding the Kanonkop baboon troop’s foraging profile to refine our interpretation of local Holocene human δ^13^C and δ^15^N values. Up to now, we have interpreted human values towards the positive ends of the isotopic spectra as indicating strongly marine diets and those towards the negative ends as indicating strongly terrestrial diets. Building on Lewis et al. [10], we ask: to what extent is it possible to refine these semi-quantitative statements to achieve quantitative information about archaeological human diets? This could best be accomplished with a model which allows for incorporation of isotope values and elemental composition of foods and humans, as well as enrichment factors for humans and accurate measures of metabolic routing. As it stands, however, our understanding of nutrient balancing and metabolic routing in humans is poor, and we are therefore not equipped to adopt this approach with appropriate rigour. The alternative, which we do here, is to use data from an appropriate analogue species and tissue (a fairly long-lived proteinaceous tissue) to constrain interpretation of simpler models.

## Materials and methods

### Study area

**Fig 1:**
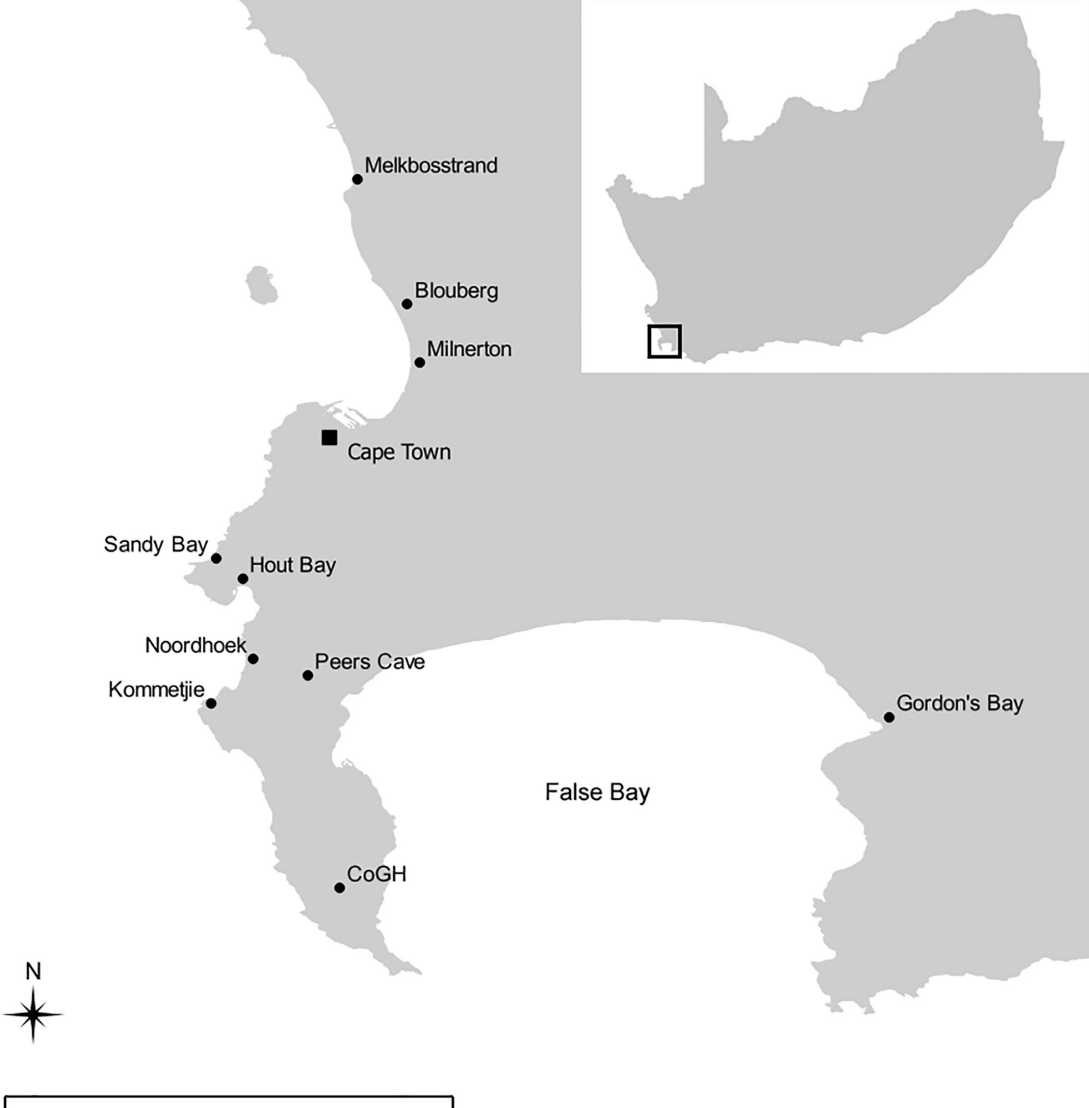
Map of study area showing locations mentioned in S1 Table (black circles). Scale bar = 20 km. CoGH = Cape of Good Hope section of the Table Mountain National Park. Inset: map of South Africa indicating location of area depicted by larger map (black square). Grey base shape file was obtained from the Municipal Demarcation Board of South Africa.

All but six of the samples in this study come from the Cape Peninsula (latitude 33°55’–34°21’ S; longitude 18°18’–18°29’ S) or immediate surrounds. We set geographic boundaries of the study region at Gordon’s Bay (34°09’ S; 18°52’ E) in the east and Melkbosstrand (33°43’ S; 18°26’ E) in the north (Fig 1) because of complications in isotope ecology further afield: terrestrial and marine organisms exhibit overlap in δ^15^N values to the north [28,70], and in δ^13^C values to the east [28,70,71]. All terrestrial plants and animals, marine invertebrates, teleosts and elasmobranchs, and three Cape fur seals (*Arctocephalus pusillus*) from which delta values were obtained were collected in the above-defined area (or off the coast in this area) [10,72–74]. We also included marine mammal (n = 3) and bird (n = 3) tissue samples collected along the coast between the Cape Peninsula and Elands Bay (32°19’ S; 18°20’ E). The taxa in question are highly mobile, and are known to travel considerable distances (>100 km) up and down South Africa’s west coast [75–80]. It stands to reason therefore that such animals found on the west coast might have ranged as far south as the Cape Peninsula and are suitable for our analyses.

The Cape Peninsula’s cool, wet winters and warm, dry summers define its temperate, Mediterranean-type climate [81,82]. Across the study area, mean annual temperatures range from 16°C to 22°C and total annual rainfall varies between ~400 mm and ~2200 mm (South African Weather Service unpublished data) [81]. Aspects of local climate have changed through time since the Last Glacial Maximum [83,84], but the general pattern of isotope ecology through the mid- to late Holocene is likely to have been similar to the present [85,86].

The study area falls within the Fynbos Biome, and includes three of the biome’s major vegetation complexes, as defined by Rebelo et al. [87]: fynbos, renosterveld and strandveld [88]. Fynbos and strandveld communities include very little grass, whereas renosterveld may take the form of shrubland or grassland with a significant shrub component [87,89,90]. Fynbos and renosterveld communities also feature substantial [edible] geophyte components, with geophyte biomass and diversity being particularly high in the latter [87,89]. Indigenous grasses in the region (found mainly in renosterveld communities) are almost all species that follow the C_3_ photosynthetic pathway [87,90,91]. C_4_ species that do occur are found mainly along watercourses or in marshy areas, and/or on relatively nutrient-rich substrates (in renosterveld) [87,90,92]. As argued elsewhere, they did not make a significant contribution to Holocene hunter-gatherer diets in the study area [93].

### Sample collection, preparation and isotope analysis

Archaeological human remains were obtained from collections curated at Iziko South African Museum and at the Department of Human Biology at the University of Cape Town, or from recent rescue excavations (S1 Table). Isotope values for these have been published previously [27,29,94,95]. All individuals in this study have been directly radiocarbon dated using bone collagen, and date to ≥ 2000 years ago, when all communities in this region were hunter-gatherers. Details regarding sample processing have been published elsewhere [96].

JCS and assistants opportunistically collected samples of marine and terrestrial animals important in archaeological human diets between 1979 and 2014. MCL and assistants collected terrestrial plant and marine invertebrate samples from the southern part of the Peninsula (south of 34°14’ S) between 2010 and 2012. The marine invertebrates collected by MCL and assistants were members of the Mollusca belonging to the classes Bivalvia (mussels) and Gastropoda (limpets). As per University of Cape Town Science Faculty Animal Ethics Committee policy, research involving molluscs other than those in the class Cephalopoda does not require ethical approval.

Initial processing of samples collected by MCL and assistants has been detailed previously [10]. For this study, however, we defatted samples by ultrasonicating each one in a 2:1:0.8 (v/v/v) methanol:chloroform:water solution for 30 minutes, and then rinsing three times with deionised water. Other values included in the dataset are from samples that were defatted following similar protocols, using either the above-mentioned solution [74] or a 1:2 (v/v) methanol:chloroform solution [72,73]. Following defatting, all elasmobranch muscle samples were rinsed in distilled water three times to remove urea [72]. This compound is found at high concentrations in marine elasmobranch tissues [97,98], and may lower apparent ^15^N/^14^N ratios of muscle tissue [99,100].

We weighed sub-samples of tissues that had not previously been analysed (~0.4 mg of animal tissue and ~2.9 mg of plant tissue) into tin cups on a Sartorius micro balance. These were combusted in a Flash 2000 elemental analyser and the resultant gases passed, via a Conflo IV gas control unit, to a Delta V Plus isotope ratio mass spectrometer (Thermo Scientific, Bremen, Germany) for stable isotope analysis. Precision of these analyses was determined using in-house standards, all of which have been calibrated against International Atomic Energy Agency standards: chocolate, valine and seal bone (for analyses of marine vertebrate tissue); chocolate, valine and sucrose (for analyses of marine invertebrate tissue); and lentil and Merck gel (for analyses of plant samples). Repeated analyses of standards yielded mean δ^13^C and δ^15^N values with standard deviations less than 0.25 ‰ in all cases. We report carbon and nitrogen isotope ratios using standard delta (δ) notation in parts per thousand (‰), relative to international standards (PeeDee Belemnite for carbon and atmospheric N_2_ for nitrogen).

### Data preparation and analyses

Given apparent collagen–keratin isotopic disparities [101], we corrected baboon hair δ^13^C and δ^15^N values from Lewis et al. [10] for comparison with human bone collagen. We did so using average ε*_collagen–keratin_ values determined for *Macaca mulatta* (−0.3 ‰ for C and +0.4 ‰ for N [102]). Similarly, we adjusted delta values of human prey animal bone collagen for use in models (S2 Table) in light of fractionation-related differences in bone collagen and muscle isotope ratios [101,103–105]. We used ε_bone collagen–flesh/muscle_ values for the taxon in question, or the best proxy for which values were available in the literature, as correction factors. To account for the Suess effect–the observed decrease in the ^13^C/^12^C ratio of atmospheric CO_2_ since 1860 [106,107]–we further adjusted all δ^13^C values of baboon hair samples and human foods less than 200 years old (S2 Table). We determined a correction factor for each year for terrestrial samples by calculating the average of all atmospheric CO_2_ δ^13^C values available from the Carbon Dioxide Information Analysis Centre [108] and Commonwealth Scientific and Industrial Research Organisation, Australia [109] for that year (using only data for full years from each station; S3 Table), and then subtracting this from −6.48 ‰ (the pre-industrial value provided by Hellevang and Aagaard [106]). Given that the magnitude of the Suess effect differs between marine and terrestrial environments [110], we multiplied the resultant values by 0.65 to generate correction factors for marine samples. We lacked %C and %N values for certain flesh samples (analysed prior to 1983) and for flesh of animals from which bone collagen was analysed. Where this was the case, we used values for the best available proxies within our dataset or from the literature (S2 Table).

We tested the bivariate normality of the human bone collagen isotope data using a generalised Shapiro-Wilk’s test for multivariate normality [111]. This test indicated that the data follow a bivariate normal distribution (MVW = 0.96, p = 0.12), so we tested for a relationship between δ^13^C and δ^15^N of these samples using a Pearson’s product moment correlation coefficient. We also ran a simple linear regression between human bone collagen δ^13^C and δ^15^N values. We used K nearest-neighbour randomization (KNNr) tests [112] to assess possible differences between bone collagen delta values of male and female archaeological humans, human bone collagen values and corrected modern baboon hair isotope values, and delta values of different food types. To gain further insight into any apparent differences between archaeological humans and baboons, we ran Wilcoxon rank sum tests on δ^13^C and δ^15^N values independently. We opted for non-parametric tests in light of the non-normal distribution of the baboon values and drastically uneven sample sizes (N_human_ = 35, N_baboon_ = 9). For the same reasons, we ran Kruskal Wallis rank sum tests to further interrogate isotopic differences between food groups. Where Kruskal Wallis tests indicated significant differences between groups, we determined which groups differed from each another using Wilcoxon’s rank sum tests with Holm corrections applied to p-values [113]. We carried out all analyses in the R statistical platform, version 3.4.3 [114].

### Stable isotope mixing models

We determined estimates of proportional contributions of different foods to archaeological human diets by means of Bayesian SIMMs [115], run using the MixSIAR package (version 3.1.7) [116]. Based on ecology and taxonomy, we divided potential foods into four source groups for use in models: terrestrial plants, terrestrial vertebrates, marine vertebrates and marine invertebrates (S2 Table). In light of uncertainty regarding trophic enrichment factors (TEF) for human bone collagen [117,118] we ran four different sets of models, incorporating different pairs of ε^13^C_collagen-diet_ and ε^15^N_collagen-diet_ values into each. This allows us to explicitly illustrate the effects of changing ε^13^C and ε^15^N values on model output. Such effects could also be elucidated through a full Bayesian analysis, which would provide a posterior probability distribution of possible TEF values. In our case, an analysis of this type would be difficult to implement however, as it would require simultaneous estimation of diet contributions and TEFs. If we had the knowledge to specify diet contributions as fixed (with error), a different analytical approach would still be required, as the MixSIAR package does not currently allow for estimation of TEF values based on other data [66]. Estimation of TEF values using Bayesian analysis was therefore beyond the scope of this study.

For our different models, we used TEF values towards the low and high extremes of values reported in the literature for humans and pigs (which are thought to be good metabolic analogues for humans [119]); +3 ‰ and +5 ‰ for δ^13^C and +3 ‰ and +6 ‰ for δ^15^N [40,118,120–123] with standard deviations of 0.5 in all values. For ease of reference, we will refer to these models as Low C-low N (ε^13^C = +3 ± 0.5 ‰ and ε^15^N = +3 ± 0.5 ‰), Low C-high N (ε^13^C = +3 ± 0.5 ‰ and ε^15^N = +6 ± 0.5 ‰), High C-low N (ε^13^C = +5 ± 0.5 ‰ and ε^15^N = +3 ± 0.5 ‰) and High C-high N (ε^13^C = +5 ± 0.5 ‰ and ε^15^N = +6 ± 0.5 ‰), hereafter. To facilitate visual assessment of the suitability of these values, we plotted minimum convex polygons (the smallest straight line-bounded polygons that incorporate the values in question) on axes with isotope values of human bone collagen and human foods.

Given that we modelled the diets of individual humans, we incorporated process error, but not residual error, into our models [124]. Initially, we ran each model with Markov Chain Monte Carlo parameters set for “very short” runs, as defined for this package (chain length = 10 000, burn-in = 5 000, thin = 5, chains = 3) [116]. Output from Gelman-Rubin [125,126] and Geweke [127] diagnostic tests for MCMC convergence suggested that none of the models reached convergence during initial runs. We therefore reran each model repeatedly, increasing run lengths until convergence had been reached–evidenced by $\hat{R}$ being < 1.05 for all variables (Gelman-Rubin test) and absolute z-scores of ≤ 5% of variables in each chain being outside ± 1.96 (Geweke test). We assessed correlations of posterior values for each final model to determine its ability to isolate contributions from different food sources–strong negative correlations between foods in close proximity in isotopic space indicate problems in this regard [62]. We set the threshold correlation coefficient value for a “strong” correlation at 0.7 [128]. Finally, we aggregated sources into terrestrial (plants and vertebrates) and marine (invertebrates and vertebrates) groups *a posteriori* [49], to ascertain estimates of total terrestrial and marine contributions to human diets.

## Results

### Isotope ratios of archaeological human foods

**Fig 2:**
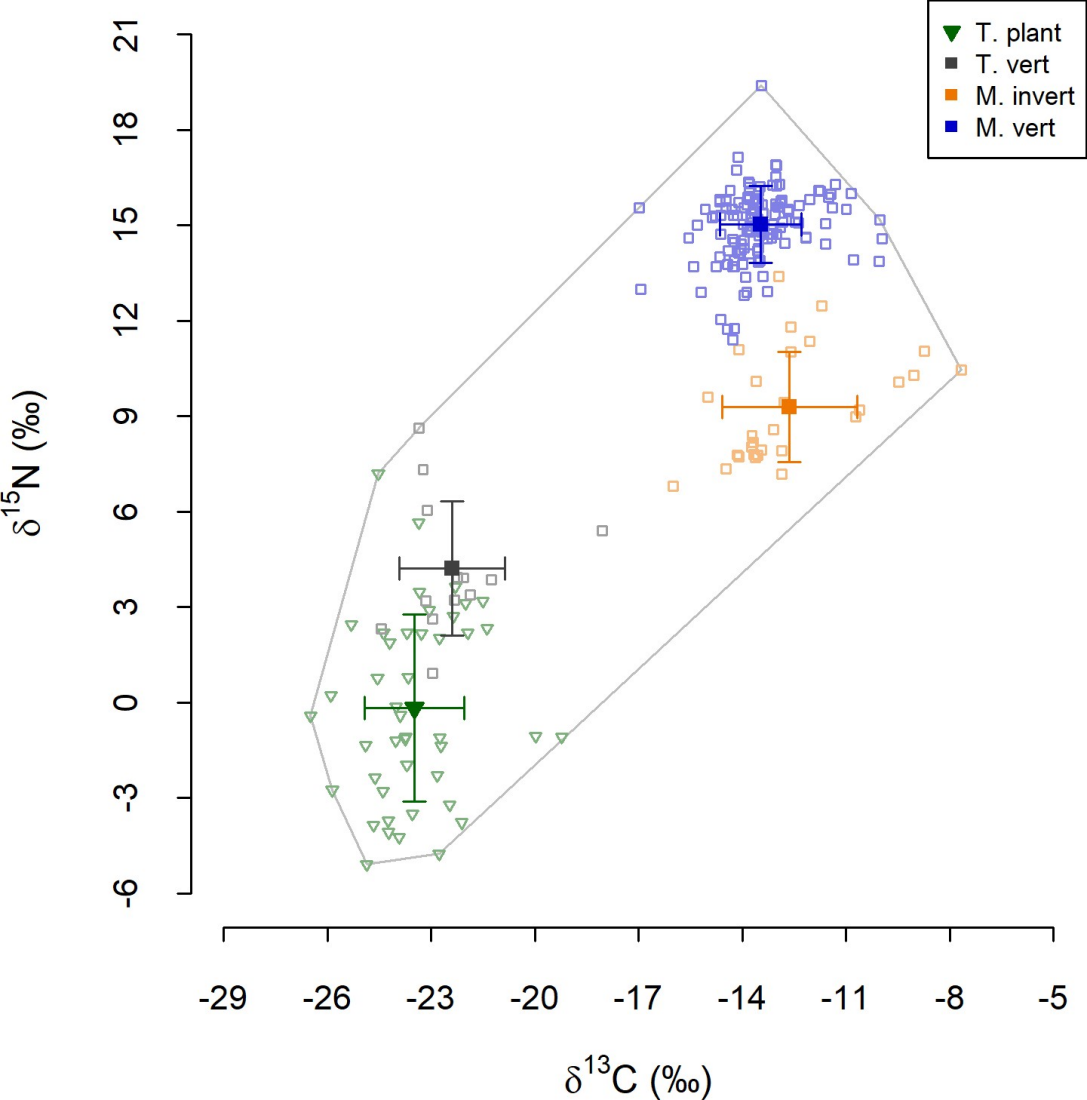
Isotope ratios of sources in models of Cape Peninsula Later Stone Age human diet. Open symbols denote raw values for individual plants and animals as indicated in the key. The grey outline indicates the minimum convex polygon around all raw values. Filled symbols and error bars show mean values and standard deviations for each group.

Across all foods, δ^13^C values ranged from −26.5‰ to −7.7‰ and δ^15^N values from −5.1‰ to 19.4‰ (Fig 2). The four food groups, namely terrestrial plants (n = 44), terrestrial vertebrates (n = 13), marine vertebrates (n = 127) and marine invertebrates (n = 29), were all isotopically distinct from one another (KNNr p < 0.01 in all cases). There were significant between-group differences in δ^13^C (χ^2^_3_ = 127.71, p < 0.001) and δ^15^N values (χ^2^_3_ = 162.92, p < 0.001), with all food groups differing from all other groups in both cases (Table 1). Isotopic differences between groups varied in magnitude. There was a marked distinction between foods of marine origin and foods of terrestrial origin in terms of δ^13^C, but smaller differences between terrestrial plants and vertebrates, and between marine invertebrates and vertebrates (Fig 2). Both marine food groups exhibited higher δ^15^N values than terrestrial groups, but the marine–terrestrial disparity in δ^15^N values was less pronounced than that in δ^13^C values. Nonetheless, results of these statistical tests suggest that these groups are appropriate for use in isotope-based models of archaeological humans from the Cape Peninsula.

**Table 1:** Results of Wilcoxon’s rank sum tests for differences in delta values of food groups.

	δ^13^C		δ^15^N	
Groups	W	p_Holm_	W	p_Holm_
Terrestrial plant–Terrestrial vertebrate	158	0.031	55	< 0.001
Terrestrial plant–Marine invertebrate	1276	< 0.001	1274	< 0.001
Terrestrial plant–Marine vertebrate	5588	< 0.001	5588	< 0.001
Terrestrial vertebrate–Marine invertebrate	377	< 0.001	361	< 0.001
Terrestrial vertebrate–Marine vertebrate	1651	< 0.001	1651	< 0.001
Marine invertebrate–Marine vertebrate	2326.5	0.031	17	< 0.001

N_Terrestrial plant_ = 44, N_Terrestrial vertebrate_ = 13, N_Marine vertebrate_ = 127, N_Marine invertebrate_ = 29.

### Isotope ratios of humans and baboons

**Fig 3:**
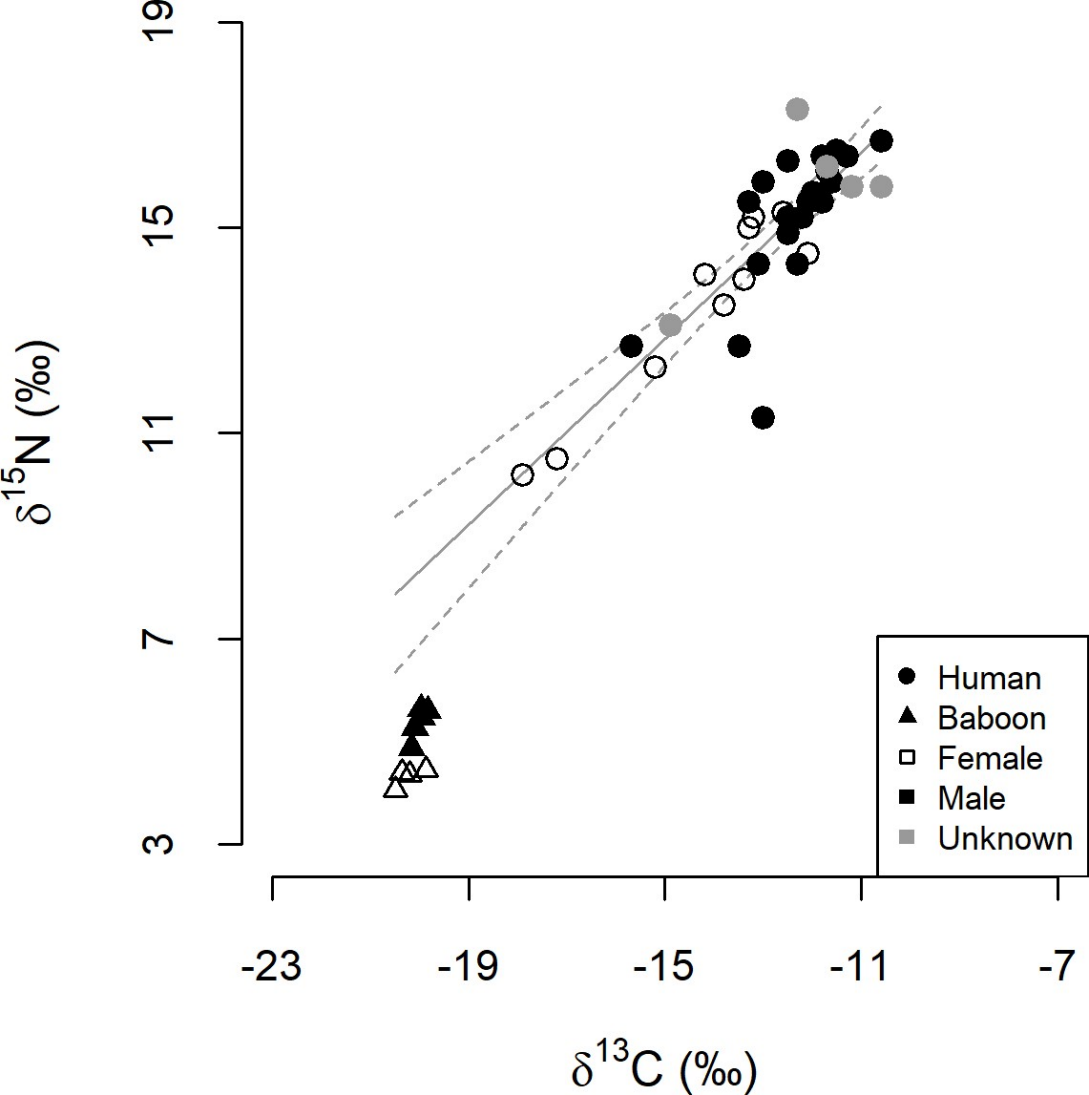
Isotope ratios of Later Stone Age human bone samples and modern baboon hair samples. Symbols denote species and sex as stated in the legend. The solid line depicts the relationship between human δ^13^C and δ^15^N values, described by *δ*^15^N = 0.9 × *δ*^13^C + 26.4 (dashed lines indicate 95% confidence intervals). The lines are extended beyond the range of human values to show how baboon values relate to the output from the regression analysis.

There was marked variation in archaeological human bone collagen delta values; δ^13^C values ranged from −17.9‰ to −10.6‰, and δ^15^N values from 10.2‰ to 17.3‰ (n = 35, Fig 3). Human δ^13^C and δ^15^N values were positively correlated (r = 0.85, p < 0.001), and regression analysis confirmed the presence of a significant linear relationship between the two (F_(1, 33)_ = 89.07, p < 0.001), described by the equation:

$$\delta^{15}N=0.9\times \delta^{13}C+26.4$$

where delta values are in parts per thousand (Fig 3). A high proportion of the ^13^C- and ^15^N-rich human samples were from males (9 out of 10 of known sex at the ^13^C- and ^15^N-rich extreme of the continuum), but the sexes were not isotopically distinct (KNNr p = 0.09).

There was a substantial isotopic disparity between human bone and corrected baboon hair samples (KNNr p < 0.001). This was driven by differences in both δ^13^C (W = 315, n_human_ = 35, n_baboon_ = 9, p < 0.001) and δ^15^N values (W = 315, n_human_ = 35, n_baboon_ = 9, p < 0.001); corrected baboon hair values were ^13^C- and ^15^N-poor, relative to human bone collagen. All baboon samples fell well below the human regression line–baboon δ^15^N values were 2.7‰ to 4.0‰ lower than values predicted by the model for humans with corresponding δ^13^C values (Fig 3).

### Trophic enrichment factors and models of human diets

**Fig 4:**
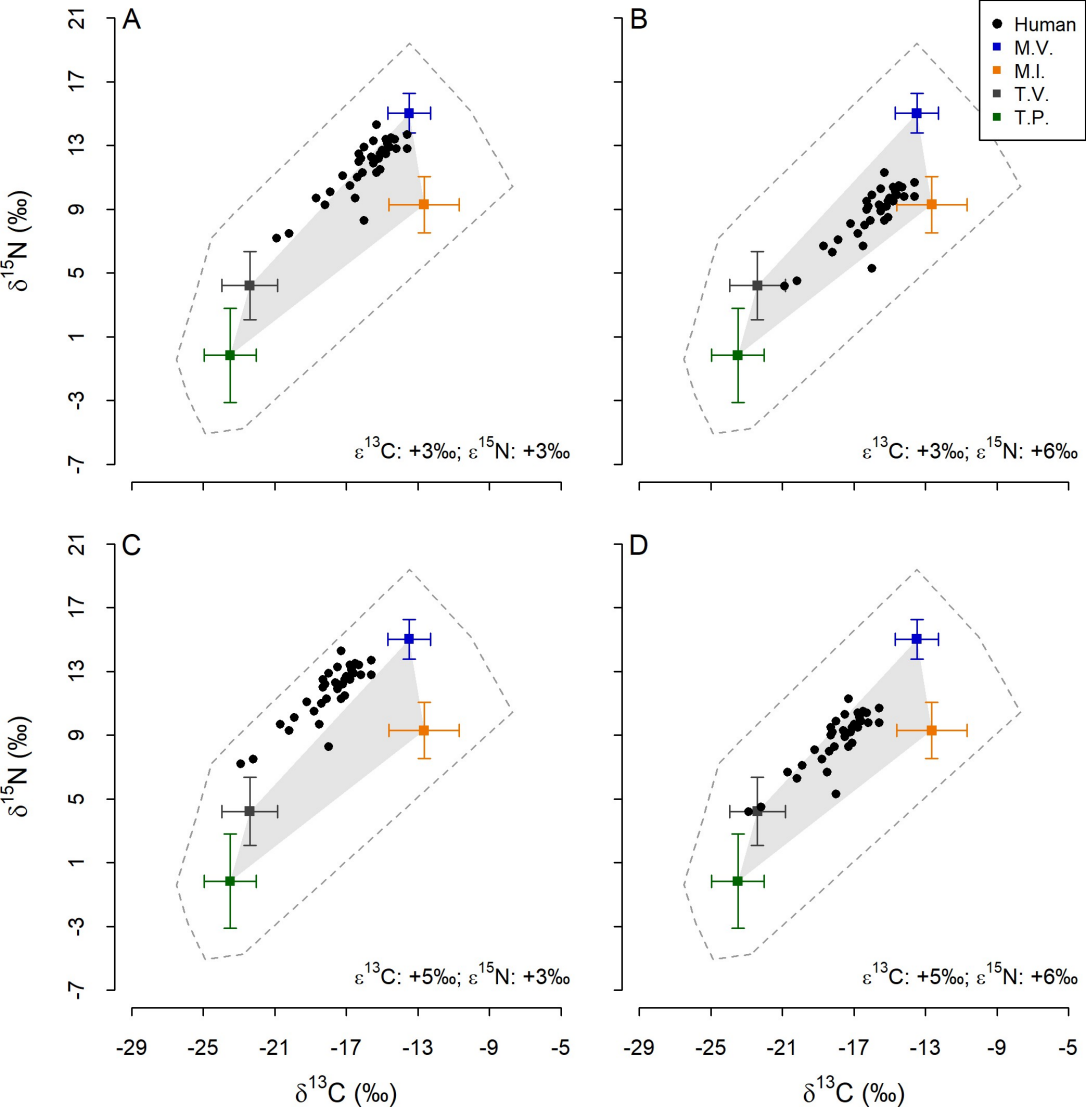
Isotope ratios of Later Stone Age humans and food groups from the Cape Peninsula. Symbols denote humans and food groups as indicated in the legend. The dashed grey outline indicates the minimum convex polygon around all raw values and the solid grey polygon the minimum convex polygon around mean values for food groups. Squares and error bars show mean values and standard deviations for each group. T.P. = terrestrial plant, T.V. = terrestrial vertebrate, M.I. = marine invertebrate and M.V. = marine vertebrate.

Irrespective of which combination of TEF values we applied, all points representing human bone collagen samples fell within the minimum convex polygon (MCP) around raw food values following correction for trophic enrichment (Fig 4). Conversely, no combination of TEF values that we used resulted in all human bone collagen points falling within the MCP surrounding mean values for different food groups. However, the numbers of human bone collagen points that fell inside this polygon varied substantially depending on the TEF values used. All but one of these points fell inside the food group mean value MCP with ε^13^C_collagen-diet_ of +3‰ and ε^15^N_collagen-diet_ of +6‰ applied, whereas all but one fell outside the same MCP with ε^13^C_collagen-diet_ of +5‰ and ε^15^N_collagen-diet_ of +3‰. The other two combinations of TEF values resulted in intermediate numbers of points falling within the food group mean value MCP.

**Fig 5:**
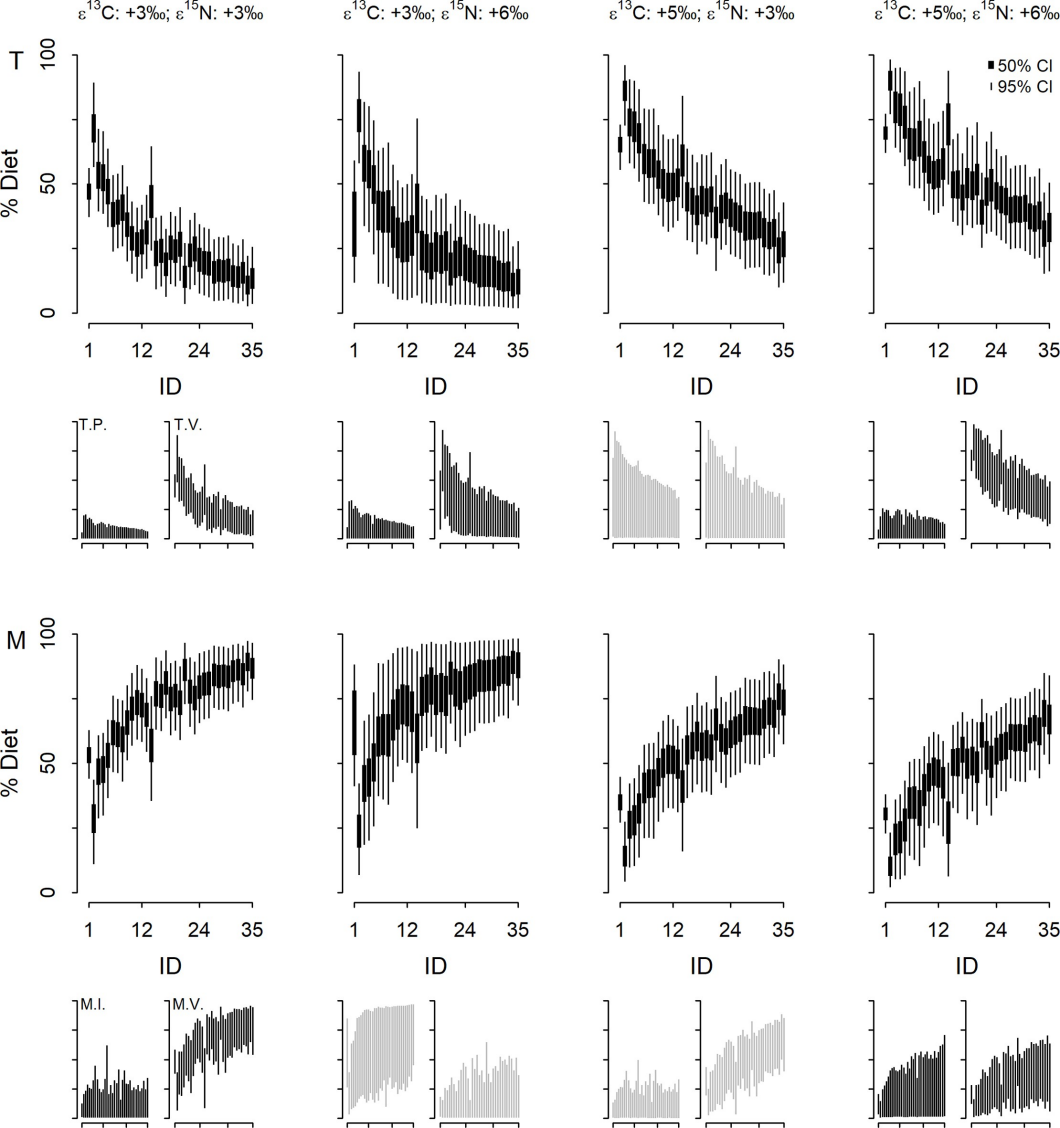
Contributions of food groups to diets of Later Stone Age humans on the Cape Peninsula. Plots in each column correspond to those in the first column labelled according to food type. Line segments in larger graphs denote credible intervals as indicated in the legend (top-right), generated using Bayesian stable isotope mixing models. Smaller graphs show only 95% CI on the same sets of axes as those in larger graphs. Grey line segments indicate that foods in that row were indistinguishable for that model. ID numbers identify individual humans, arranged in order of δ^13^C values–increasing from left to right. T = terrestrial foods, T.P. = terrestrial plant foods, T.V. = terrestrial vertebrate foods, M = marine foods, M.I. = marine invertebrate foods and M.V. = marine vertebrate foods.

Aggregated estimates of dietary contributions from all four models showed marked inter-individual variation in marine–terrestrial breakdown of human diets (Fig 5). Across all model outputs, there was a general pattern of increasing marine contributions and decreasing terrestrial contributions, with increasing δ^13^C values of human bone samples. The specific contribution estimates yielded by models with different TEF values varied, however. The High C-high N model yielded lower upper and lower limits of 95% credible intervals (CI_95%_) than any other model, with lower limits from this model ranging from 2% to 53%, and upper limits from 23% to 85%. The Low C-low N model yielded the highest lower limits of CI_95%_ (ranging from 11% to 78%) and upper limits of CI_95%_ that were higher than those of the High C-low N and High C-high N models, but generally slightly lower than those of the Low C-high N model.

Model performance diagnostics indicate that two of the models were unable to distinguish between certain food groups defined *a priori* (Fig 5). In the case of the Low C-high N model diagnostics, dietary contributions of marine invertebrate and marine vertebrate foods were indistinguishable (r = −0.78). The High C-low N model was unable to reliably differentiate between contributions of terrestrial plant and terrestrial vertebrate foods (r = −0.99), or between marine invertebrate and marine vertebrate foods (r = −0.73).

The three models that were able to distinguish between the two terrestrial food types (Low C-low N, Low C-high N and High C-high N) indicated that the humans in question obtained little of their protein from terrestrial plant foods (Fig 5). The upper limits of CI_95%_ for plant foods generated by these models were < 33% for all individuals, and < 25% for most individuals–all but five according to the Low C-high N model and all but three according to the High C-high N model. There was consensus across these models regarding minimum plant contributions to dietary protein, with lower limits of CI_95%_ for plant foods being 0% for all individuals in all models.

Outputs from the Low C-low N, Low C-high N and High C-high N models exhibited considerable inter-individual variation in maximum credible proportions of dietary protein obtained from terrestrial vertebrates (Fig 5). The differences between maximum and minimum upper limits of CI_95%_ across individuals yielded by a given model ranged from 54% to 69%. In these three models, there was a general pattern of decline in upper limits of terrestrial vertebrate CI_95%_ with increasing δ^13^C values (ID numbers match the ascending order of δ^13^C). All three model outputs show at least one individual (seven in the case of the High C-high N model) exhibited CI_95%_ upper limits > 85%. Conversely, across these model outputs, at least four individuals (26 in the case of the Low C-low N model) exhibited CI_95%_ upper limits lower than 50%. These same models indicated that no individual’s CI_95%_ included 0%. However, the Low C-high N model indicated that lower limits of terrestrial vertebrate CI_95%_ for 27 individuals were below 5%.

The Low C-low N model yielded consistently low upper limits of marine invertebrate CI_95%_; these values were < 35% for 31 out of 35 individuals in both models. The High C-high N model yielded higher CI_95%_ upper limits (26 individuals exhibited values > 40%) with a general pattern of increasing CI_95%_ upper limits with increasing δ^13^C values. Outputs from these two models indicate that all CI_95%_ lower limits were extremely low: 0% for all individuals but one according to the Low C-low N model and ≤ 1% for all individuals but one according to the High C-high N model.

The Low C-low N and High C-high N model outputs showed similar patterns of generally increasing marine vertebrate CI_95%_ upper limit values with increasing δ^13^C values. The Low C-low N model yielded markedly higher values however–only one individual’s CI_95%_ upper limit for marine vertebrate foods was < 50%. Again, both models showed general patterns of increasing CI_95%_ lower limits with increasing δ^13^C values, but the values themselves, and the width of CI_95%_ differed. The higher values for marine vertebrate CI_95%_ lower limits were those yielded by the Low C-low N model; none of these values were < 6% and all but three were ≥ 20%. In the High C-high N model output, 24 of 35 CI_95%_ lower limits were > 5%.

## Discussion

Model diagnostics indicate that from a statistical point of view, all models presented here provide plausible estimates of terrestrial and marine contributions to the diets of Late Holocene humans on the Cape Peninsula. Two of the models further provide reliable estimates of dietary contributions of sub-groups within the marine and terrestrial components: terrestrial plants and -vertebrates, and marine invertebrates and -vertebrates. Outputs from all models showed similar marked inter-individual variation in diet composition (at the level of terrestrial and marine foods), but the ranges of credible intervals varied across models. CI_95%_ maxima and minima for marine foods varied by up to 13% across models–maxima ranged from 85% to 98% across models for the individual with the highest values (ID 34). Though striking, such variation is in fact not hugely meaningful, given the magnitude of measurement error relative to ranges of food delta values–the variation is comparable to the error in terms of scale. A more noteworthy difference across model outputs, is the lack of overlap of 50% credible intervals (CI_50%_) generated by models with different ε^13^C values for all but one individual. This indicates that choice of ε^13^C has a major impact on model output. Based on model outputs alone, we cannot infer which ε^13^C value is more appropriate and, hence, which of the model outputs might represent the closest approximation to the truth.

We hoped to use the baboon data and inferences from those data to constrain the interpretation of the models as applied to archaeological humans, enabling us to disregard outputs from less plausible models. However, direct comparison of humans and baboons in term of δ^13^C and δ^15^N in combination is seemingly ill-advised. As shown in Fig 3, the baboons do not fall on the same marine–terrestrial regression line as the humans; baboons are poorer in ^15^N than we would predict humans with the same δ^13^C values to be. Considering trophic enrichment in nitrogen isotopes and the higher trophic levels of vertebrates, particularly in the marine environment [129,130] (Fig 2), baboons probably fall below the regression line because they do not include marine and terrestrial vertebrate tissues in their diets [10,11,13], as humans did [31,32,34,35]. Given the observed pattern, and its likely explanation, we maintain that δ^15^N values of humans and baboons are not directly comparable.

Comparison of δ^13^C values only still seems viable, given that the ranges of δ^13^C for foods consumed by humans and baboons were similar. The δ^13^C value of Human ID 2 (the individual with the lowest δ^13^C value) was ≥ 1.9 ‰ greater than those of all baboons. From this, we infer that even humans at the low end of the ^13^C/^15^N continuum consumed more marine food than the baboons of the Kanonkop troop. The High C-high N model yields total marine food CI_95%_ for four of the most ^13^C/^15^N-depleted individuals that show some overlap with those produced by isotope-based models of baboon diets [10]. In the case of human ID 2, roughly half of the CI_95%_ overlapped with the CI_95%_ for baboons. Given the disparity in δ^13^C values, this overlap seems highly improbably. We therefore posit that the High C-high N model presents erroneous estimates of dietary inputs for the humans in question.

We also question the validity of the High C-low N model, but for a different reason. Following correction using the relevant ε values, all human bone collagen samples fell within the MCP defined by all foods, but only one fell inside the MCP defined by the food group mean values–many were well outside this envelope (Fig 4C). In contrast, TEF values used in the Low C-low N (Fig 4A) and Low C-high N (Fig 4B) models result in the majority of points (all but one in the case of the Low C-high N model) falling inside the MCP defined by the mean values for different food groups. In light of our careful isotopic characterisation of food groups, we conclude that the combination of TEF values incorporated into the High C-low N model was inappropriate [49,131].

ε^13^C_collagen-diet_ of +5‰ is unsuitable in this context. This is consistent with previous findings regarding ε^13^C_collagen-diet_ values for pigs–the best non-primate physiological analogue for humans on which controlled-feeding studies have been carried out. These studies have revealed that collagen from pigs on most diets exhibits δ^13^C values < 4.5‰ greater than those of their diets [40,104,123]. Our “low” value meets this criterion but might be slightly lower than the true value(s), given that it is towards the low extreme of the range published in the literature (by design). TEFs might in fact be slightly different for different individuals within the sample population, depending on each one’s diet composition and nutrient status [39,132–137]. In light of this, and the arguments above, we proceed with discussion of outputs from the Low C-low N- and Low C-high N models, which we believe represent the best estimates of diet composition for this population.

Only the Low C-low N model was able to reliably distinguish between marine invertebrate and -vertebrate diet components, and showed that for all except the most ^13^C and ^15^N-depleted individuals, marine vertebrates made a greater contribution than marine invertebrates. Had we used an intermediate ε^15^N value here (as discussed above), this model might have yielded less extreme invertebrate: vertebrate ratios in the marine component of the diets. A more substantial invertebrate contribution would be more consistent with the abundant limpet and mussel shells in Holocene coastal shell middens in this area [31,34].

Both the Low C-low N and Low C-high N models indicate that all humans in this sample certainly consumed some marine food, and that marine foods made up more than 18% of the modelled diets of most individuals–only one individual (ID 2) may have consumed less (as little as 7%). ^13^C and ^15^N-rich individuals (ID 27–35) consumed a heavily marine-biased diet, with marine foods comprising at least 65%, and possibly as much as 98%, of the modelled diets of these individuals. We note that the most positive values in our study are very similar to those of the most specialised marine foragers known, from the northwest coast of North America and the Arctic. Tauber [138,139] reported δ^13^C values of −12.6 and −13.0 ‰ for two 18^th^ century East Greenlanders known to have subsisted almost entirely on seafoods. By comparing their apparent ^14^C ages with their historic ages he was able to deduce that 90–95% of the carbon in their bone collagen derived from marine foods. Other archaeological and anthropological studies of high-latitude marine specialists report bone collagen δ^13^C values of up to about −13 ‰ [140,141]. It is unclear to what extent small differences in the positive end-points of δ^13^C ranges reflect regional differences in the isotopic values of foods, or differences in the extent of reliance on marine foods. These high estimates of marine protein consumption could all be inflated, but this seems unlikely. Comparison of isotopic and behavioural data for Peninsula baboons that feed on small amounts of marine foods does not support the suggestion that marine foods are “over-represented” [37] in δ^13^C and δ^15^N of proteinaceous tissues in omnivorous consumers eating marine protein-rich foods in combination with terrestrial foods rich in carbohydrates [10].

Given our approach to sample preparation (including a lipid extraction step), this study does not consider the possible contribution of carbon from lipids in the diet to bone collagen. Doing so would however have been incompatible with our analytical approach. Since most lipids contain no nitrogen, they cannot be included in a bivariate model based on C and N. The most important lipid-rich food items for the humans in question would have been whale and seal blubber; terrestrial wild animals are very lean and terrestrial plant tissues are typically lipid-poor food items. Our model outputs may therefore, if anything, underestimate the overall importance of marine foods in the diet.

## Conclusions

- In the coastal hunter-gatherers studied here, the trophic enrichment factor (TEF) for δ^13^C in human bone collagen is closer to +3 than +5‰.
- Our results less clearly constrain the TEF for δ^15^N, but we note that a value of +6‰ (rather than +3‰) results in the largest number of points (all but one) falling into the minimum convex polygon around mean values for different foods groups.
- Model outputs confirm that the wide range of human δ^13^C and δ^15^N values (7.3 and 7.1 respectively) result from the consumption of very varied proportions of marine and terrestrial foods.
- Terrestrial plant foods made only a limited contribution to the diet: the limits of CI_95%_ for plants were 0–33% for all individuals, and 0–25% for most (30/35 according to the low C–high N model).
- Low C-low N and Low C-high N model outputs combined indicate that in the most ^13^C and ^15^N-rich individuals (δ values of ~−11 ‰ and ~+17 ‰ respectively), 65–98% (95% CI) of bone collagen derived from marine foods.
- Conversely, in ^13^C and ^15^N-poor individuals (δ values of ~−18 ‰ and ~+10 ‰ respectively), 7–44% (95% CI) of bone collagen derived from marine foods.
- Attempts at quantitative dietary reconstructions for omnivores eating complex mixed diets should be framed in the light of uncertainties such as those shown above. This applies equally to attempts to calibrate radiocarbon dates on mixed marine–terrestrial consumers.
- Depending on the research questions, SIMMs might or might not be useful tools for dietary reconstruction in archaeological humans.

## Supporting information

S1 Table Bone collagen delta values from Late Holocene humans from the Cape Peninsula, South Africa. (XLSX)

S2 Table Delta values of plant- and animal tissues included in models of Cape Peninsula human diet. (XLSX)

S3 Table δ^13^C-CO_2_ values used to calculate Suess effect correction factors for plant and animal samples. (XLSX)
